# Echocardiographic Hemodynamic Monitoring in the Critically Ill Patient

**DOI:** 10.2174/157340311798220485

**Published:** 2011-08

**Authors:** Francisco J Romero-Bermejo, Manuel Ruiz-Bailén, Manuel Guerrero-De-Mier, Julián López-Álvaro

**Affiliations:** 1 Intensive Care Unit, Critical Care and Emergency Department. Puerto Real University Hospital, Cádiz, Spain; 2 Intensive Care Unit, Critical Care and Emergency Department, Medical-Surgical University Hospital of the Jaen Hospital Complex, Jaén, Spain; 3 Intensive Care Unit, Critical Care and Emergency Department. Nuestra Señora de Valme University Hospital, Sevilla, Spain

**Keywords:** Echocardiography, hemodynamic, monitoring, shock, critically ill patient.

## Abstract

Echocardiography has shown to be an essential diagnostic tool in the critically ill patient's assessment. In this scenario the initial fluid therapy, such as it is recommended in the actual clinical guidelines, not always provides the desired results and maintains a considerable incidence of cardiorrespiratory insufficiency. Echocardiography can council us on these patients' clinical handling, not only the initial fluid therapy but also on the best-suited election of the vasoactive/inotropic treatment and the early detection of complications. It contributes as well to improving the etiological diagnosis, allowing one to know the heart performance with more precision. The objective of this manuscript is to review the more important parameters that can assist the intensivist in theragnosis of hemodynamically unstable patients.

## INTRODUCTION

The critically ill patient is under an intense threat for his life. Therefore, the diagnostic methods used in the Critical Care and Emergency departments must be fast, reliable and reproducible to assure a successful therapeutic strategy.

**Image 1:**
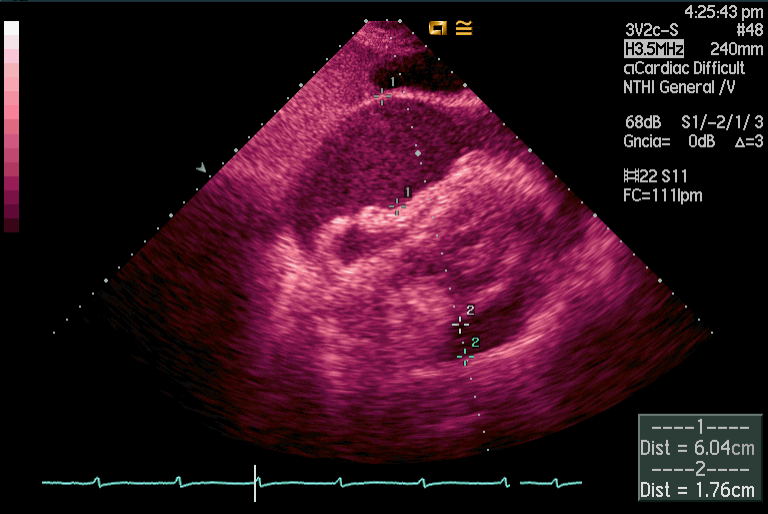
Severe pericardial effusion in subcostal transthoracic view.

**Image 2:**
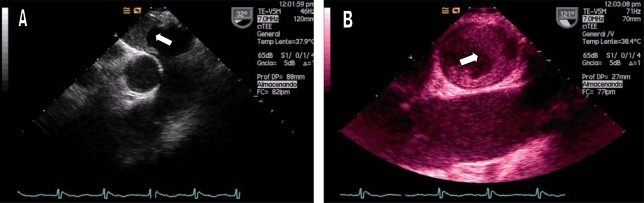
Patient in shock state, with high suspicion of septic shock due to cholecystitis (fever, right hypochondrium pain). In the transesophageal study we found a giant thrombi in the right pulmonary artery (RPA). A) TEE for 30°. B) TEE for 120°.

Echocardiography is one of the best diagnostic tools that the intensivist has, because it can be performed at the bedside**, **avoiding patients' displacements, and can provide transcendental information on real time for making vital decisions in a noninvasive or semiinvasive form, such as fluid therapy continuity, early vasoactive or inotropic treatment, realization of a pericardiocentesis in a cardiac tamponade (Image **1**), systemic fibrinolysis in severe pulmonary embolism (Image **2**), or the indication of cardiac surgery for the existence of mechanical complications in context of acute coronary syndrome, between others. In spite of this, it continues being an underused diagnostic method, even in the coronary units [1]. 

In Intensive Care Units (ICUs), the first echocardiographies were performed by cardiologists who were consulted for the diagnosis of several cardiovascular diseases. In the 1980s, French and Danish intensivists began to utilize this technique as a more global form of hemodynamic study [2]. The use of echocardiography in the ICUs increased slowly during the 1990s, when some ICUs abandoned the use of the pulmonary artery catheter (PAC). The ICU of the *Ambroise Paré Hospital *was the pioneer in the use of echocardiography for hemodynamic monitoring*.* This event, possibly, started the “*revolution” *of the echocardiography at the field of the Critical Care and Emergency. Jardin *et al*. [3] in 1994, demonstrated with transthoracic echocardiography (TTE) that the PAC, considered the best system of hemodynamic monitoring at the bedside, did not estimate correctly the left ventricular end-diastolic pressure (LVEDP). In 1998, Benjamin *et al*. [4], found similar results with TEE. In recent years advances in echocardiography are having an exponential growth.

It has been demonstrated to the scientific community that echocardiography is not an exclusive cardiologist's diagnostic method and that the intensivist is totally capacitated to perform and interpret an echocardiographical study and, unlike the cardiologist, encompassing it with the patients´ hemodynamic and respiratory parameters: a “goal directed“echocardiography. In 2005, Manasia *et al*. [5] demonstrated than the intensivists, after a 10 hour TTE course, could correctly interpret severe pericardial effusions and the left ventricular volume. These findings influenced the patients assistance, modifying their treatments in more than 35%. Recently, in a prospective study with critically ill trauma patients, it was demonstrated that the focused rapid echocardiographic evaluation (FREE) modified the plan of care in more than 50 % [6].

Most of the indications for echocardiography were recently approved by the American College of Cardiology Foundation Appropriate Use Criteria Task Force [7]. In Table **1** we summarized the main indications for echocardiography in Critical Care settings. 

In hemodynamically unstable patients, TTE should be the first choice. In any case, this study must be goal-directed (Focused Ultrasound and Echocardiography – FUSE -), because the time compels, and integrated with the patient's clinical status [8]. Once hemodynamic stability is achieved, a more exhaustive study is recommended. When poor-quality images are obtained or there are suspicions of aortic disease, valvular prosthesis dysfunction, or even for previous evaluation of electric cardioversion, transesophageal echocardiography (TEE) would be indicated. In sedated patients receiving mechanical ventilation, TEE seems easier to perform, however precaution must be taken because this condition increases the risk of esophageal perforation or an accidental extubation.

Many studies tried to compare the effectiveness of invasive and noninvasive hemodynamic monitoring methods. Within the complications related to PAC's use are the development of pulmonary artery rupture, hematoma, endocarditis, venous thrombosis, bacteriemia, arrhythmias and even death (0.4 %) [9]. The TEE is not exempt of possible complications. The most frequent are hemodynamic instability, oropharyngeal and/or esophageal injury, accidental extubation, and ventilatory changes, with low incidence (less than 1/1000). Min *et al*. [10] studied a cohort of patients who required TEE during a 10-year period. They performed up to 10,000 studies and found a very low incidence of complications: one case of hypopharyngeal perforation (0.01%), 2 cases of cervical esophageal perforation (0.02%), and no cases of gastric perforation or death.

Therefore, if the quality of the TTE images were optimal, it is reasonable to think that TTE is the best method of hemodynamic monitoring. In a classic study by Kaul *et al*., the authors compared the execution times between echocardiographic study and PAC implantation and interpretation. They found that with TEE an average of 19 minutes were spent and with PAC an average of 63 minutes [11]. However, we must remember that echocardiography is a discontinuous diagnostic method, so it is better to conclude that invasive and noninvasive methods are complementary. 

Esophageal Doppler monitoring (EDM) is a relatively easy procedure that measures blood flow velocity in the descending thoracic aorta (70% of cardiac output) using a flexible probe, similar in size to a nasogastric tube, inserted into the patient’s esophagus. This technique has the ability to estimate cardiac output and ejection time, requiring no calibration, minimal training and having a very good safety profile. Usually, the probe is uncomfortable in awake patients and, for this reason, it has been mainly studied in sedated patients. EDM also has limitations: the use of a correction factor that isn´t constant (K-factor), since it depends on hemodynamic status; and the Doppler beam must be within 20º of axial flow to obtain a correct measurement of aortic blood flow [12]. Nonetheless, EDM seems like an attractive technique alternative to invasive hemodynamic monitoring systems.

In this review we describe the essential parameters for hemodynamic monitoring in the critically ill patient guided by echocardiography.

## PRELOAD OPTIMIZATION

In patients with severe sepsis or septic shock, early optimization of cardiac output through intensive fluid therapy has been shown to reduce morbidity and mortality. It is also known that not all patients respond equally and that excessive fluid therapy can lead to cardiopulmonary failure [13]. 

Traditional parameters estimating blood volume, closely related to preload, central venous pressure (CVP) or pulmonary artery systolic pressure (PASP) have not been proven reliable in predicting fluid responsiveness [14]. However, echocardiography is presented as an alternative non-invasive or semi-invasive (in the case of the TEE), which may offer useful parameters to determine the critical patient’s preload such as ventricular volume changes, respiratory changes in inferior vena cava (ICV) diameter (with TTE) or superior (with TEE), or also respiratory changes in aortic flow velocity.

### Ventricular Walls Study

The visualization of left ventricular systolic obliteration ("*kissing papillary muscles sign*") may be indicative of hypovolemia and thus present a need for intensive fluid therapy. However, the presence of uni-or biventricular dilation does not exclude the need of such treatment. In severe hypovolemia, cases of ventricular pseudohypertrophy have been observed.

### Respiratory Changes in Cava Veins Analysis and Stroke Volume Variability

In mechanically ventilated patients with positive end-expiratory pressure (PEEP), measurement of CVP, pulmonary capillary wedge pressure (PCwP) or right ventricular end-diastolic diameter can bias the analysis of fluid responsiveness because intrathoracic pressure is increased in these cases, and therefore may decrease venous return and change stroke volume. However, several studies with TEE have shown that stroke volume variability (SVV) or respiratory variability of the superior vena cava (SCV) diameters can faithfully reproduce fluid therapy responsiveness in these patients. To calculate the SCV collapsibility index TEE is necessary to obtain the maximum and minimum diameters in M-mode or two-dimensional. The SCV collapsibility index is calculated as the difference in maximum and minimum diameter divided by the first and multiplied by 100. A result above 36% indicates the need for fluid to increase cardiac output with a sensitivity of 90% and a specificity of 100% [15]. 

**Image 3:**
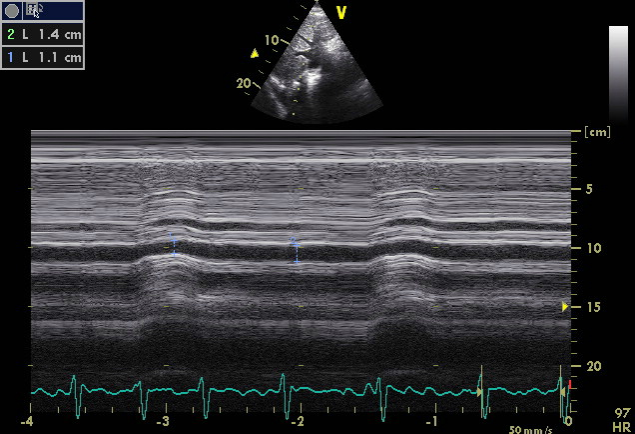
ICV collapsibility index calculation in a patient with urological severe sepsis. Applying the equation: [(maximum diameter - minimum diameter) / maximum diameter] x 100, we get a rate of 21.4%, which suggests the need to start with vasoactive treatment.

If a TEE probe isn’t available, it is possible to guide volume expansion with TTE analyzing, in subcostal view, the ICV diameters. In contrast to SCV, the ICV is not affected by intrathoracic pressure but by an increased intraabdominal pressure. These diameters tend to increase with inspiration unless hypovolemia is present. The ICV collapsibility index (Image **3**) is calculated using the same formula as that of the SCV. Compliance rate exceeding 15% (12% and 17% depending on the series), may indicate the need for volume expansion with a sensitivity and specificity of 90%. Mechanical ventilation (which causes high prevalence of dilated ICV) and various conditions that increase abdominal pressure (abdominal surgery, pancreatitis), would limit the usefulness of this parameter [16-18].

**Image 4:**
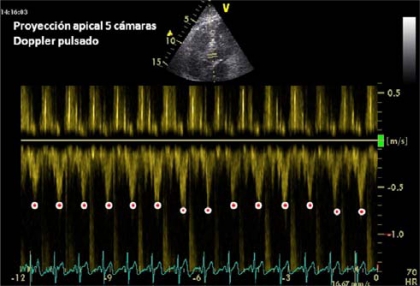
Stroke volume maximum velocity determination (pulsed Doppler in LVOT). This image was collected immediately after a load of 500 cc of 0.9% Saline in a patient with septic shock of biliary tract origin. There was no evidence of significant variability compared to basal imaging, suggesting the need for vasoactive treatment.

The incorporation of Doppler studies has positively influenced the hemodynamic monitoring with echocardiography. Pulsed Doppler in Left Ventricular Outflow Tract (LVOT) (Image **4**), and descending thoracic aorta with TEE (delta Vpeak) in patients with mechanical ventilation, can give us information on the variability of stroke volume respiratory changes and predict fluid responsiveness [19]. A variation of more than 12% predicts response to fluid challenge (defined as an increase in cardiac output by at least 15% with a standard fluid bolus) with high sensitivity and specificity.

### Parameters Derived from the Study of Left Ventricular Diastolic Function (LVDF)

**Image 5A-5B:**
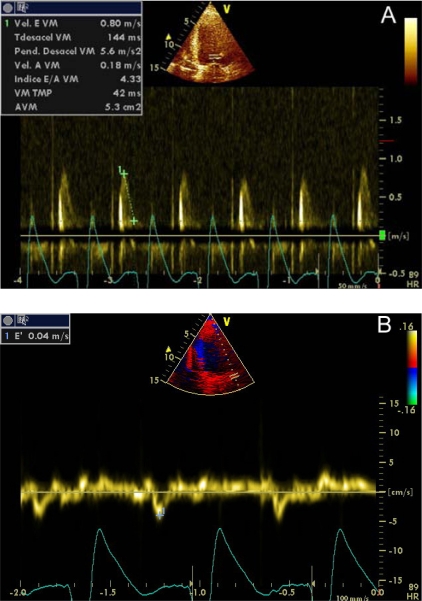
Third degree diastolic dysfunction (restrictive pattern). Transmitral pulsed Doppler (4) and Doppler Tissue Imaging (B). E/E´ ratio was 20.

The study of LVDF provides important information about the performance of LV. Restrictive pattern (third degree diastolic dysfunction), which occurs when LV compliance is limited, usually is associated with high LVEDP [20]. Restrictive filling pattern is characterized to represent a normal or decreased isovolumetric relaxation time, a marked increase in the mitral peak Doppler E-wave, a reduction in deceleration time and a very low mitral A wave velocity (Images **5A-5B**).

Alternative methods to assess diastolic function have been proposed which aren´t preload-dependent and therefore are more sensitive to demonstrate early response to fluid therapy, such as Tissue Doppler Imaging (DTI) or Color M-mode Doppler flow propagation velocity (PV) [21-23].

**Image 6A-6B-6C:**
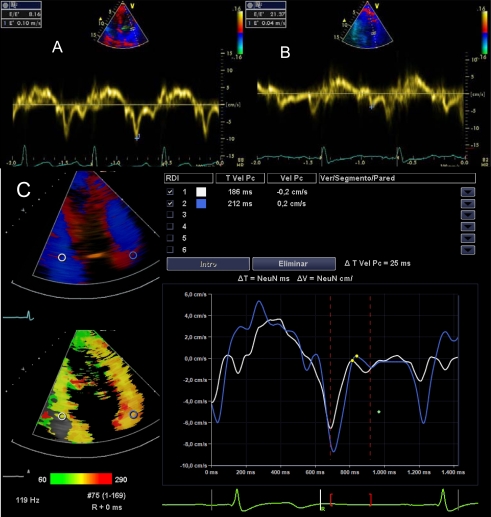
E/E´ratio. (**A**) Normal, (**B**) elevated, (**C**) E/E´ estimation with advanced echocardiographic software.

DTI is a Doppler mode that detects the speed of contraction of myocardial tissue. The early mitral filling velocity (E)/early diastolic mitral annular velocity (E') ratio (E/E'), which combines the influence of transmitral pressure and myocardial relaxation, has been shown to predict LVEDP. For E ' wave calculus, it is necessary the average of measurements in the lateral and septal mitral walls, which can be done sequentially or directly if you have advanced echocardiographic software (Images **6A-6C**). However, in patients in shock, preferences in the location where to measure the E´wave have not been demonstrated. An E/E 'ratio less than 8 accurately predicts a normal LVEDP and when this ratio is greater than 15, is related to very high LVEDP. When this ratio is intermediate (8-15), there are controversies. The E/E´ ratio has proven to be a good predictor of left ventricular dysfunction in experimental models of of mechanical ventilation weaning failure and a prognostic factor in septic and critically ill patients in general [24,25]. PCwP can be estimated with Nagueh formula [26]: 1.9 + (1.24 x E/E '). This parameter is equivalent to the mean atrial pressure and, therefore, to LVEDP. The velocities obtained with DTI can be influenced by cardiac motion (translation, torsion and rotation), and even blood flow. These effects can be minimized with the use of a smaller simple size.

**Image 7:**
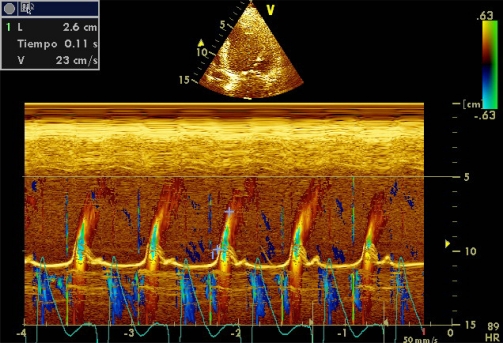
Color M-mode Doppler flow propagation velocity.

Color M-mode Doppler flow propagation velocity (PV) is altered in subjects with diastolic dysfunction. In these patients, mitral E-wave amplitude decreases and flow moves more slowly toward the left ventricular apex. Unlike pulsed Doppler E wave, PV is relatively independent of atrial pressure. Values less than 50 cm/s are associated with significant diastolic dysfunction (Image **7**). In patients with LV systolic dysfunction, a E/PV ratio ≥ 2.5 can estimate a PCwP> 15 mmHg [27]. However, the applicability of the PV is limited because it depends on left ventricular volume and sharpness of the event.

**Image 8:**
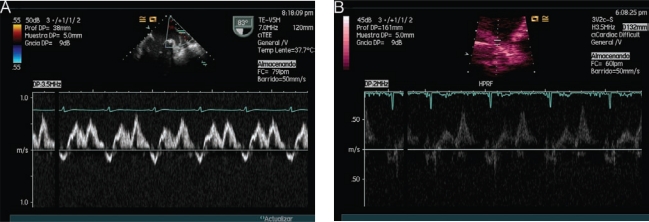
Diastolic function study: Pulsed Doppler in pulmonary veins. **A**) TEE image, **B**) TTE image. In both, systolic wave < diastolic wave.

Another way to study left ventricular preload is by pulmonary venous flow analysis (Images **8A**, **8B**). Using this, we can estimate the left atrial pressure (LAP), obtained by Kuecherer´s equation [28]: LAP = 35 - (0,39 × SFF), where SFF (Systolic Filling Fraction) is derived from the ratio of the adition of systolic and diastolic VTI, adding diastolic VTI and multiplying by 100. Values above 15 mmHg are considered pathological. Gorcscan *et al.* [29], proposed another formula to calculate LAP in patients with congestive heart failure: Systolic Arterial Pressure – 4 (mitral insufficiency peak)², obviously this equation is subject to mitral insufficiency.

### Passive Leg-raising

Passive leg-raising (PLR) (30 degrees) is a reversible maneuver that mimics rapid volume expansion, with strong predictive value. It has been studied in patients with hypovolemia, severe sepsis and pancreatitis [30]. The test is considered positive when there is an increase in stroke volume up to 12% [31]. This maneuver has been validated in numerous studies with patients in spontaneous breathing and in a study with patients under mechanical ventilation. In patients with intra-abdominal hypertension (> 16 mmHg), PRL seems to be responsible for false negatives [32].

## ANALYSIS OF VENTRICULAR SYSTOLIC FUNCTION

Patients with refractory shock to initial fluid therapy require an analysis of ventricular function prior to initiation of vasoactive or inotropic therapy.

The estimation of the biventricular systolic function by echocardiography is part of the comprehensive management of patients with hemodynamic instability. Basic competence in echocardiography for intensivists proposed by Cholley *et al.* [33], is defined as the ability to visually differentiate a normal and moderately or severely depressed LV systolic function. This visual assessment can be crucial for the hemodynamic stabilization of patients in shock. When shock is associated with severe left ventricular dysfunction, the initiation of inotropic therapy may increase survival. If left ventricular ejection fraction (LVEF) was above 40% (cut-off value proposed in most studies), the vasoactive treatment of choice for the moment and although some studies associated with increased incidence of myocardial dysfunction [34], is norepinephrine.

### LV Systolic Function

The best known form of objectifying LV systolic function is through the LVEF calculus, which is the percentage of end-diastolic volume that is ejected, but this parameter has several limitations. LVEF is clearly influenced not only by LV contractility, but also by the state of preload and afterload that are frequently altered in patients with shock; however, LVEF has proved to be a very sensitive measurement of changes in contractility when ventricular function is impaired. The usual method for calculating LVEF is the modified Simpson, although in an experienced operator, the subjective estimation of contractility is sufficient to orient towards the need for inotropic therapy.

**Image 9:**
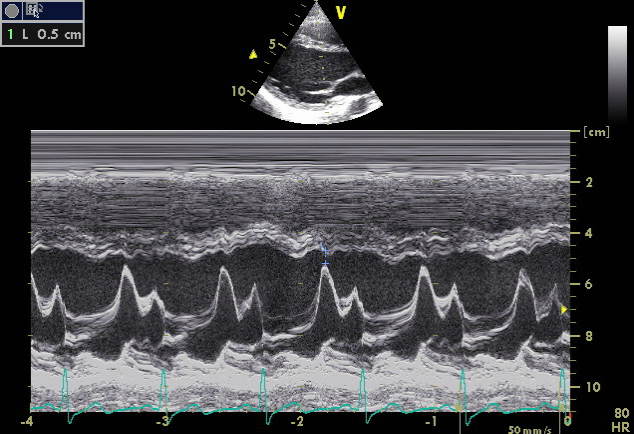
E-interventricular septum distance.

**Image 10:**
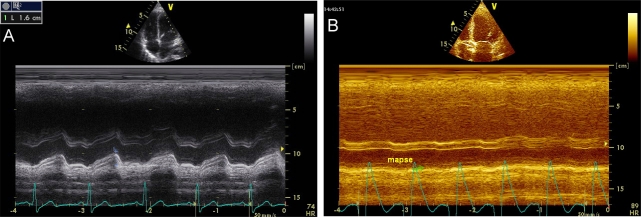
Mitral Annular Plane Systolic Excursion. **A**) Normal, **B**) Severe systolic dysfunction.

The study of left ventricular systolic function with M-mode echocardiography has lost its initial importance due to the development of other more specific methods. The calculation of LVEF by Teicholtz method is less appropriate when there are segmental contractility disturbances, described mainly in ischemic heart disease but also in sepsis-induced myocardial dysfunction [35]. Other measurements are still useful are to calculate the distance between mitral E wave and the interventricular septum (IVS) and the MAPSE (Mitral Annular Plane Systolic Excursion). The E-IVS is measured in parasternal long axis at the mitral valve level and when its value is greater than 1 cm it is associated with severe systolic dysfunction (Image **9**). The MAPSE is quantified in apical 4-chamber view on the lateral mitral annulas; values less than 1 cm are also associated with systolic dysfunction (Image **10**).

**Image 11A-11B:**
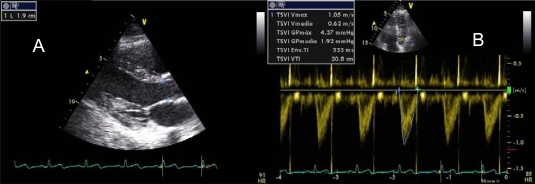
Stroke volume calculus. Applying the continuity equation SV = π x R² x VTILVOT, SV = 3.1415 x 0.95 cm² x 20.8 cm = 59 ml.

The cardiac output (CO) is the most important hemodynamic parameter in critically ill patients and can be easily estimated by echocardiography. To calculate it, we need to get the diameter of the LVOT in proto-midsystole, in parasternal long axis, and calculate the VTI in apical 5-chamber view with pulsed Doppler (Image **11A**-**11B**). Applying the equation π x R² x VTILVOT, where R = radius of the LVOT in cm, we get the stroke volume (SV), with normal values of 45 ± 13 ml/m². With this parameter we can make several derived calculations [SV Index (SVI) = SV x Body Surface Area, Cardiac Output = SV x heart rate, cardiac index = CO x Body Surface Area].

**Image 12:**
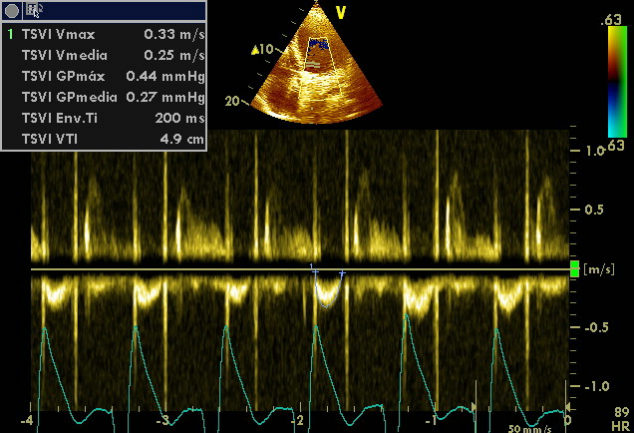
VTI in LVOT in a patient with cardiogenic shock.

If the quality of the image obtained for calculating the diameter of the LVOT in parasternal long-axis view is not optimal, it is possible to determinate it in apical 3-chamber view and, alternatively, a measurement of 2 cm in adult can also be used empirically. If the patient is in atrial fibrillation, an average of at least 6 determinations should be performed [36]. The determination of LVOT diameter is subject to intra-and interobserver variability. By decomposing the formula for obtaining the SV, we note that the first two factors are always constant and therefore the value of the VTILVOT is the main component, the only one which should be calculated after the initial study. VTILVOT normal values are between 18 and 20 cm, values less than 12 cm are highly suggestive of shock status (Image **12**). We must not forget that all these calculations are altered in aortic valve disease or significant subaortic obstruction.

Tei index [37] is an echocardiographic parameter that measures the global cardiac function (systolic and diastolic). Its determination is through transmitral and LVOT pulsed Doppler or DTI in lateral and septal mitral walls. The Tei index has been widely circulated within the echocardiography community of Japan and the U.S., it is simple to obtain, reproducible and independent of heart rate and blood pressure. It is defined as the sum of isovolumetric contraction time and isovolumic relaxation time divided by ejection time. Normal values are between 0.37 ± 0.05. Several studies have correlated Tei index with clinical severity of heart failure in patients with dilated cardiomyopathy, cardiac amyloidosis, and after an acute myocardial infarction.

### Right Ventricular Systolic Function

In certain clinical cases, a right ventricular (RV) dysfunction may be responsible for the continuing situation of shock. The right ventricle can also be studied with echocardiography with ability to provide information on RV function similar to that provided by the PAC. Size, wall thickness, contractility, ejection fraction (RVEF) and abnormal movements of the interventricular septum (IVS) can be studied. The RVEF is usually estimated qualitatively, because the shape of the RV is not cylindrical, unless there is 3D echocardiography or radionuclide angiography available, although there are currently various parameters that can estimate the RVEF with a strong correlation.

The abnormal movements of the IVS and the flattening or paradoxical motion may suggest the existence of elevated right ventricular end-diastolic pressures. The leftward shift of IVS during systole is a sign of pressure overload and during diastole is a sign of volume overload, both reflecting an abnormal RV hemodynamics [38].

**Image 13:**
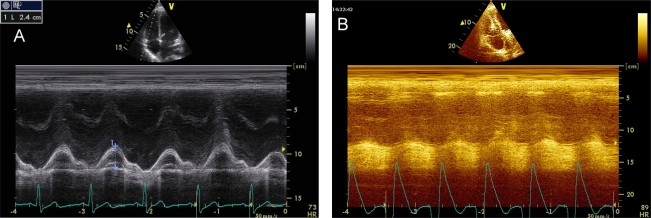
TAPSE. **A**) Normal, **B**) RV systolic dysfunction.

Tricuspid Annular Plane Systolic Excursion (TAPSE), the RV Tei index, tricuspid DTI and Cardiac Magnetic Resonance Imaging have gained value. The longitudinal displacement of the tricuspid annular plane toward the apex of the RV (*Tricuspid annulus Plane Systolic Excursion*) is measured in 4-chamber view with M-mode on the lateral wall of the tricuspid annulus. A value of less than 1.6 cm is suggestive of RV systolic dysfunction. This parameter has shown a high correlation with RVEF calculated by radionuclide angiography [39] (Image **13A**-**13B**).

The RV Tei index can be calculated in a similar form to that described for the LV or by DTI. Normal values are slightly lower than those of LV, 0.28 ± 0.04, and its importance lies in the overall estimate of right heart function, both systolic and diastolic. The determination in RV-DTI of a S wave less than 10 cm/s is indicative of right ventricular systolic dysfunction.

In contrast to LV function, the right ventricle usually has a high afterload in sepsis due to increased pulmonary vascular resistance due to the appearance of acute lung injury or acute respiratory distress syndrome (ARDS), which can lead to a decrease in right ventricular cardiac output. In addition, the RV does not usually compensate acute elevations in afterload, since it is perfused in diastole and systole as well and may develop cardiogenic shock because it is unable to expand quickly, which occurs in massive pulmonary thromboembolism. 

The two main causes of acute pulmonary hypertension in the ICUs are massive pulmonary embolism and ARDS (up to 25% of cases). In both, there is RV dilatation, leading to diastolic overload, and dyskinesia of the IVS, producing systolic overload. In these patients it is essential to reduce RV afterload to ensure coronary perfusion pressure.

**Image 14:**
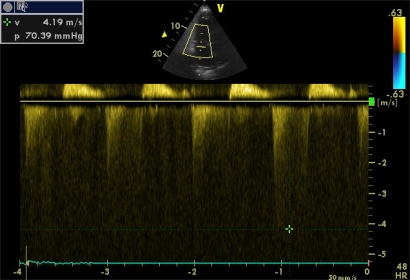
Pressure gradient between RV and right atria in a severe pulmonary hypertension.

Doppler echocardiography can estimate systolic pulmonary artery pressure (SPAP) with good correlation with invasive methods. For its calculation, the Bernoulli equation is applied (4 x velocity ²) on a jet of tricuspid insufficiency, a condition *sine qua non*, thus obtaining the pressure gradient between RV and right atria (Image **14**). In the absence of pulmonary stenosis or other obstruction in the RV outflow tract, right ventricular systolic pressure is equal to that of the pulmonary artery, therefore, the SPAP is calculated by adding, to the pressure gradient, the mean right atrial pressure (MRAP). A transpulmonar flow in “W”, determined with pulsed Doppler in paraesternal short axis view, in suggestive of severe pulmonary hypertension.

**Image 15:**
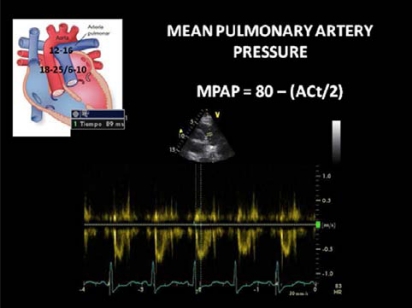
Mean pulmonary artery pressure estimation. (ACt = Aceleration Time).

All patients with hemodynamic instability usually have a central venous access, hence the determination of the CVP may be possible to determine the MRAP. But everyone knows that the CVP has its limitations, thus in most studies the MRAP is considered a continuous variable between 5 and 20 mmHg. Other studies have shown that the MRAP can be estimated more accurately (Table **2**) analyzing the changes in the ICV respiratory diameters. Pulmonary hypertension is considered when SPAP is greater than 35 mmHg and when it is greater than 60 mmHg is it considered severe. Mean and diastolic pulmonary artery pressures can also be calculated with echocardiography (Image **15**).

In a not too distant future, the intensivist will be trained in new echocardiographic technologies. These will include the DTI, myocardial deformity analysis, speckle tracking, 3D and real-time 3D. So, we could do a safe theragnosis and will be able to investigate our critically ill patient´s heart performance. We probably will think not only in the Frank Starling law of the heart, but also in the Torrent Guasp law [40]. 

## AFTERLOAD ESTIMATION

The second classic parameter for determining global tissue perfusion, systemic vascular resistance (SVR), can also be determined by echocardiography if we had previously the MRAP, with the following formula: SVR = (MAP - Diastolic arterial pressure / CO) x 79.9 [41]. Another possible way to estimate afterload is calculating the systolic wall stress; however, this calculation is of little use and acceptance in clinical practice.

## CONCLUSIONS

In a hemodynamically unstable patient, echocardiography can quickly provide enough information to get a successful theragnosis. Its use in critically ill patients is unquestionable; however, no clinical trials that endorse an increase in survival with echocardiography-guided therapy have been performed.

In pursuit of "excellence" in the treatment of critically ill patients, intensivists were trained and successfully performed percutaneous tracheostomy, thoracocentesis, pericardiocentesis, continuous renal replacement therapies, fibrobronchoscopies, transitory and permanent pacemakers, among others. Now is the time that hemodynamic monitoring guided by echocardiography be generalized in all ICUs.

## Figures and Tables

**Image 16:**
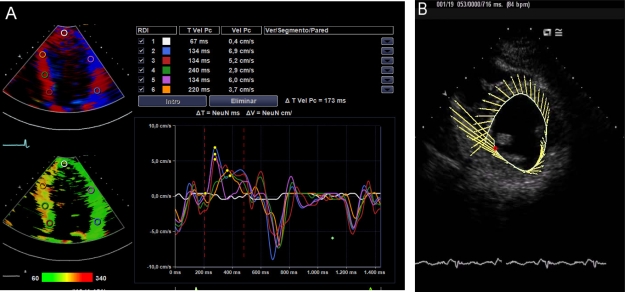
New echocardiographic techniques. **A**) Advanced DTI, **B**) Speckle tracking.

**Table 1.** Main Indications of Echocardiographic Study in Critical Care Settings.

TTE	Hypotension or hemodynamic instability. Assessment of volume status. Acute chest pain with suspected myocardial infarction and nondiagnostic complementary tests. Suspected complication of myocardial infarction. Evaluation of biventricular systolic function during acute coronary syndrome. Respiratory failure or hypoxemia of uncertain etiology. Pulmonary embolism: Diagnosis and to guide therapy. Severe deceleration injury or chest trauma. Suspicion of valvular or structural heart disease. Initial evaluation of suspected infective endocarditis. Percutaneous noncoronary cardiac procedures: Guidance for pericardiocentesis, septal ablation, or right ventricular biopsy.
TEE	Poor quality of TTE images. Suspected acute aortic pathology. Evaluation of valvular structure and function to assess an intervention. Prosthetic valve dysfunction. To diagnose infective endocarditis. Assessment to cardioversion, and/or radiofrequency ablation.

**Table 2.** Mean right atrial pressure according to respiratory changes in inferior cava vein.

Inferior Cava Vein diameter	Inspiratory colapse	Right Atria Pressure (mmHg)
≤2.1 cm	>50%	3 (R:0-5)
	< 50%	8 (R:5-10)
>2.1cm	>50%	
	<50%	≥15
